# Correction

**DOI:** 10.1080/21505594.2024.2363658

**Published:** 2024-06-09

**Article title**: Clinical features and risk factors for pyogenic liver abscess caused by multidrug-resistant organisms: A retrospective study

**Authors**: Q Long, X Zhao, C Chen, M Hao and X qin

**Journal**: *Virulence*

**DOI**: https://doi.org/10.1080/21505594.2024.2356680

This article was published earlier with an error in the ‘Table 1’. This has now been corrected and republished in the original article. The below mentioned is the updated Table 1 which is now corrected in the original article.

**Table 1.** Baseline characteristics of the study patients (N = 239)

Characteristic	Value
Age (years), mean ± SD	56.6 ± 14.6
Hospitalization days (day), mean ± SD	26.3 ± 24.5
Abscess size (mm), mean ± SD	66.1 ± 31.2
Sex (male), n (%)	168 (70.3)
Antibacterial drug use within 6 months before onset, n (%)	52 (21.8)
Hospitalization within 6 months before onset, n (%)	78 (32.6)
Origin, n (%)	
Cryptogenic	108 (45.2)
Direct dissemination	47 (19.7)
Biliary origin	71 (29.7)
Hematogenous dissemination	13 (5.4)
Comorbidities, n (%)	
Diabetes	83 (34.7)
Hypertension	74 (31.0)
Cirrhosis/hepatic failure	21 (10.5)
Malignant tumor	57 (23.8)
Cardiovascular disease	9 (3.8)
Chronic renal insufficiency	4 (1.7)
Cerebrovascular disease	11 (4.6)
Abscess location, n (%)	
Right lobe	160 (66.9)
Left lobe	40 (16.7)
Bilateral	39 (16.3)
Number of abscesses, n (%)	
Single	164 (68.6)
Two or more	75 (31.4)
Pathogen Quantity, n (%)	
Single species	204 (85.4)
Two species	30 (12.6)
Three species	5 (2.1)
Treatment, n (%)	
Antimicrobial agents alone	74 (31.0)
Antimicrobial agents and drainage	144 (60.2)
Antimicrobial agents and surgery	21 (8.8)

